# Tyrosine kinase 4 is involved in the reproduction of the platyhelminth parasite *Schistosoma japonicum*

**DOI:** 10.1186/s13071-017-2453-5

**Published:** 2017-10-18

**Authors:** Han Ding, Fengchun Liu, Lulu Zhu, Fei Wu, Quan Liu, Siyu He, Wei Shao, Yinan Du, Cuiping Ren, Jijia Shen, Miao Liu

**Affiliations:** 1 Department of Microbiology and Parasitology, Anhui Provincial Laboratory of Microbiology and Parasitology, School of Basic Medical Sciences, 81#Meishan Road, Hefei, Anhui 230032 People’s Republic of China; 2 Anhui Key Laboratory of Zoonoses, Anhui Medical University, 81#Meishan Road, Hefei, Anhui 230032 People’s Republic of China

**Keywords:** *Schistosoma japonicum*, Reproduction, SjTK4, Piceatannol

## Abstract

**Background:**

Schistosomiasis is one of the most common parasitic diseases affecting millions of humans and animals worldwide. Understanding the signal transduction pathways and the molecular basis of reproductive regulation in schistosomes is critically important for developing new strategies for preventing and treating these infections. Syk kinases regulate the proliferation, differentiation, morphogenesis, and survival of various types of cells and have been identified in invertebrates. Tyrosine kinase 4 (TK4), a member of the Syk kinase family, plays a pivotal role in gametogenesis in *S. mansoni*, affecting the development of the testis and ovaries in this parasite. The role of TK4, however, in the reproduction of *S. japonicum* is poorly understood*.*

**Methods:**

Here, the complete coding sequence of *TK4* gene in *S. japonicum* (*SjTK4*) was cloned and characterized. The expression of *SjTK4* was analyzed at different life-cycle stages and in various tissues of *S. japonicum* by qPCR. Piceatannol, a Syk kinase inhibitor, was applied to *S. japonicum* in vitro. The piceatannol-induced morphological changes of the parasites were observed using confocal laser scanning microscopy and the alterations in important egg-shell synthesis-related genes were examined using qPCR analyses.

**Results:**

*SjTK4* mRNA was differentially expressed throughout the life-cycle of *S. japonicum*. *SjTK4* mRNA was highly expressed in the ovary and testis of *S. japonicum*, with the level of gene expression significantly higher in males than in females. The expression levels of some important egg-shell synthesis related genes were higher in the piceatannol-treated groups than in the vehicle-treated control group and the number of eggs and germ cells also decreased in a concentration-dependent manner. Importantly, large pore-like structures can be found in the testis and ovaries of males and females after treating with piceatannol.

**Conclusion:**

The results suggest that SjTK4 may play an important role in regulating gametogenesis of *S. japonicum*. The findings may help better understand the fundamental biology of *S. japonicum*. Moreover, the effect of *S. japonicum* treatment by piceatannol provides us with a new idea that inhibition of SjTK4 signaling pathway can effectively retard the development of the testis and ovaries*.*

## Background

Schistosomiasis is a serious parasitic disease present in certain tropical regions worldwide, with a conservative estimate of more than 200 million affected people [1]. Schistosomiasis in humans is associated with three major parasites: *Schistosoma haematobium*, *Schistosoma mansoni* and *Schistosoma japonicum*. In China, *S. japonicum* is responsible for one of the most serious parasitic diseases in humans [2, 3]. Praziquantel had been effective in the treatment of schistosomiasis. Although praziquantel-resistant schistosomiasis has been reported [4], this is not a problem in current MDA treatment protocols. The important position of PZQ for the treatment of schistosomiasis, and an anticipation of extremely increased usage in future, create a fear of schistosomes becoming resistant to it globally [5]. Moreover, reliance on a single drug to treat schistosomiasis globally is not an effective approach for parasite control, so there exists a desperate need for alternatives. The schistosome life-cycle is complex, involving multiple developmental stages. After *S. japonicum* infects a definitive host, the female’s reproductive system matures to produce eggs subsequent to the pairing between male and female worms. Females must pair with a male in order to undergo complete sexual development and the production of infectious eggs. After pairing with a male, the female reproductive structures, mainly the ovary and the vitelline glands, undergo terminal differentiation [6]. However, understanding the significance of the development and fundamental regulations in the reproductive biology of schistosomes, as well as in the pathogenic consequences of schistosomiasis, is still in its initial stage. Transmission of schistosomiasis depends on the release of eggs from the definitive host. Soluble egg antigens released by the miracidia within the eggs is the major cause of the pathology from the infection [7, 8]. Due to the importance of eggs for the life-cycle and for inducing pathogenesis, many genes related to the reproduction, including *Vasa* [9], *Sj79* [10], and *Wnt5* [11], have been studied in an effort to control reproductive development. Therefore, understanding the signal transduction pathways and the molecular basis of reproductive regulation in schistosomes is critically important for developing new strategies for preventing and treating schistosomiasis. Moreover, a better understanding of the fundamental biology of flatworms is also of broad scientific interest.

Tyrosine kinase 4 (TK4) is a member of the spleen tyrosine kinase (Syk) family [12]. The N-terminus of the Syk protein contains a pair of Src homology 2 (SH2) domains that are connected to each other by a linker and separated from the catalytic domain by another longer linker [13].

In vertebrates, Syk belongs to the Src family of non-receptor tyrosine kinases, which associate directly with surface receptors, including B-cell receptors and Fcγ receptors, and participate in a variety of signal transduction pathways [14]. Syk kinases are expressed in cells of the hematopoietic system and play specialized roles in inflammatory processes [15]. Syk kinases also play an important role in regulating the proliferation, differentiation, morphogenesis and survival of various types of cells [16–19]. Syk-related kinases have also been identified in invertebrates. A single Syk-related kinase, SHARK, is found in *Drosophila melanogaster* [20]. Syk-related kinases have also been found in the polyp (*Hydra vulgaris*) and sponges [21]. However, *Caenorhabditis elegans* does not contain any Syk-related tyrosine kinases genes. A single Syk-related molecule, SmTK4, is found in *S. mansoni*; it plays a pivotal role in gametogenesis and affects the development of the testes and ovaries of *S. mansoni* [22]. Thus, the roles of the *TK4* gene of *S. japonicum* (*SjTK4*) in the reproduction of this schistosome species warrant exploration.

In the present study, it has been confirmed that the *SjTK4* gene was expressed in *S. japonicum* during all developmental stages both in females and, at a higher level, in males. In particular, the *SjTK4* gene was highly expressed in male testes and female ovaries. To further explore the role of SjTK4 in *S. japonicum*, we used confocal laser scanning microscopy (CLSM) to examine the effects of piceatannol, a Syk kinase-specific inhibitor that has been previously investigated as a potential compound against schistosomes [22], on *S. japonicum*. Treatment of piceatannol led to significant morphological changes in the testes and ovaries of *S. japonicum*, as well as alterations in the mRNA levels of egg-shell formation-associated genes, including *P14*, *P48*, *egg-shell precursor* and *FS800*. Our results were consistent with previous findings in *S. mansoni* and further confirmed that the *SjTK4* gene also plays an important role in regulating reproductive organ development in *S. japonicum*.

## Methods

### Animals and parasites

Female Kunming mice (6 weeks old) and New Zealand rabbits (3 ± 0.5 kg) were purchased from the Experimental Animal Center of Anhui Province in Hefei, China. The mice were housed under specific pathogen-free conditions at Anhui Medical University. The rabbits were fed in individual cages at 19–29 °C, with free access to food and water. *Oncomelania hupensis* snails infected with *S. japonicum* (a Chinese mainland strain) were provided by the Hunan Provincial Institute of Parasitic Diseases in China. To collect cercariae, a total of 50 *Oncomelania hupensis* snails infected with *S. japonicum* were exposed to light for 3–4 h (25–28 °C), and cercariae collected in a centrifuge tube and placed on ice for 15 min. Cercariae were concentrated by centrifugation at 800× *g* for 5 min. Mice (*n* = 20) were infected with 60–80 cercariae, and rabbits (*n* = 3) with approximately 3000 cercariae. To collect *S. japonicum* eggs, liver tissue from rabbits 6 weeks post-infection was homogenized and then subjected to consecutive fractional filtration. The filtrate was centrifuged. The supernatant and tissue-containing layers were removed, leaving the egg-containing layer, which was diluted in 1.2% saline and passed through a nylon net (300 mesh per inch) [9]. Parasites were harvested at 16, 24 and 42 days after infection. Adult male and female parasites (at 24 and 42 days) were separated by gentle brushing. All parasites were subsequently washed three times with phosphate-buffered saline (PBS; pH 7.4) and cryopreserved at -80 °C.

### Molecular cloning of *SjTK4*

According to the de novo transcriptome assembly of *S. japonicum* from GenBank with the project accession number of PRJNA343582 [23], we obtained information about complete sequence of the *SjTK4* gene. Primers were designed and synthesized by Sangon Biotech Co. Ltd. (Shanghai, China). The forward and reverse primers were 5′-ATG AAT GTG ACT AAT AAT GTG GTA GTA ACT CCA-3′ and 5′-TTA AAA TGA TAT TCC ATC ACT ACC ACC A-3′, respectively. For PCR amplification, a 50 μl total reaction volume was prepared containing 0.2 μg cDNA, 5 μmol of each primer, 0.5 mmol dNTPs and 2.5 U high-fidelity DNA polymerase (Thermo Scientific). After a denaturation step at 94 °C for 5 min, thermal cycling was performed as follows: 94 °C for 45 s, 60 °C for 1 min, 72 °C for 1 min for 30 cycles and a final extension at 72 °C for 8 min. The PCR products were purified by agarose gel electrophoresis and sent for sequencing to Sangon Biotech Co. Ltd. (Shanghai, China). The sequencing result was uploaded to the National Center for Biotechnology Information (NCBI) database (GenBank accession number: KX984125).

### Amino acid sequence comparisons analysis and phylogenetic analysis of *SjTK4*

The full-length cDNA sequence of *SjTK4* was submitted to online analysis with BLASTX and ORF Finder in NCBI (http://www.ncbi.nlm.nih.gov/). Expasy (http://web.expasy.org/compute_pi/) [24] was used to calculate molecular weight and isoelectric point (pI) of SjTK4. Multiple sequence alignments of SjTK4 with TK4s from other organisms were performed using DNAman. EMBOSS needle (global alignment) (http://www.ebi.ac.uk/Tools/psa/emboss_needle/) [25] was used to analysis pairwise sequence alignments. InterProScan (http://www.ebi.ac.uk/interpro/search/sequence-search) [26] was used to analyze the primary domains of the SjTK4 protein. To confirm which family SjTK4 belonged to, phylogenetic analysis was performed based on the amino acid sequence similarity of the catalytic domain of TK4. A total of 23 sequences belonging to the Syk/Zap70 and Src families were selected for the bioinformatics analysis. All sequences were downloaded from the NCBI database (http://www.ncbi.nlm.nih.gov/) and integrated into one file for further analysis. A phylogenetic tree was constructed using MEGA5.05 software [27]. The Neighbor-Joining method and Kimura 2-parameter model were used to estimate the evolutionary history for major clades. The reliability of phylogenetic tree was evaluated by the bootstrap analysis (1000 replicates). A cut-off value less than 50% will not be displayed. The final phylogenetic tree was obtained after editing using the graphic resources contained in the FigTree software and exported in PDF format.

### qPCR of *SjTK4*

To detect *SjTK4* gene expression, female and male parasites were cut into four parts: testis, non-testis, ovary and non-ovary [28]. Total RNA was extracted from the testis, non-testis, ovary, non-ovary, schistosomula (day 16), and adult female and male parasites at different stages (days 24 and 42) using TRIzol® Reagent (Life Technologies, Carlsbad, CA, USA). RNA was quantified and used the same amount to construct the different cDNA using a PrimeScript RT reagent kit (Takara, Dalian, China). Real-time quantitative reverse transcription PCR (qPCR) was performed in duplicate using 1 μl of cDNA from each sample and SYBR Premix Ex Taq II (Takara, Dalian, China) according to the manufacturer’s instructions. All reactions were performed using an ABI-Prism StepOnePlus™ Real-Time PCR System (Foster City, CA, USA) with the following conditions: 95 °C for 10 s, followed by 40 cycles of 15 s at 95 °C and 40 s at 60 °C followed by 15 s at 95 °C, 1 min at 60 °C and 95 °C for 15 s, the threshold cycle (Ct) values were determined using default threshold settings. The Ct values were defined as the fractional cycle number at which the fluorescence passes the fixed threshold, which were analyzed and generated directly by StepOne software. Primers for qPCR were designed and synthesized by Sangon Biotech Co. Ltd. The PCR primers for *SjTK4* were 5′-CCT TGC TGA AGC ACG TAC AA-3′ and 5′-GCC AAT TCA AGG ACA AGC AT-3′. The PCR primers for *PSMD4* were 5′-ACT TTG AAC AGG AGA TGG CGA-3′ and 5′-GCC TCA GGA CAA CGG AAC C-3′.

The relative quantification levels were calculated using the 2^(−ΔΔCT)^ method [29]. The endogenous gene *PSMD4* (26S proteasome non-ATPase regulatory subunit 4) maintains stable expression levels during the various developmental stages [30] and was thus selected as the internal reference control.

### In vitro culture of adult schistosomes

Adult schistosomes were harvested after perfusion and washed three times with M199 medium before being cultured in vitro with M199 (Catalogue number: 12340–030 Gibco, Grand Island, NY, USA) at 37 °C and 5% CO_2_. The M199 medium was supplemented with an antibiotic/antimycotic mixture (1.25%, Catalogue number: 15240–096 Gibco) and fetal calf serum (10%, Catalogue number: 10099–141, Gibco). For each experiment, 15 pairs of *S. japonicum* were maintained in a 6-well plate in 3 ml of culture medium.

### Piceatannol treatment and morphological analysis

Piceatannol (3, 4, 39, 59-tetrahydroxy-trans-stilbene; Selleck Chemicals, Shanghai, China) was dissolved in dimethyl sulfoxide (DMSO). In each experimental group, 15 adult couples of *S. japonicum* were cultured in 3 ml of medium and treated with one of three different concentrations of piceatannol (35 mM, 70 mM, or 100 mM). The medium with inhibitor was changed every 24 h for 7 days. During this time, the viability of parasites, worm pairing, and the number of eggs in the culture medium were recorded. For the morphological analysis, *Schistosoma* worm pairs were fixed in a solution of alcohol (95%), formalin (3%), and glacial acetic acid (2%) (AFA) for at least 24 h. Parasites were stained in hydrochloric acid-carmine dye (Ourchem, Shanghai, China) for 17 h, and then destained in acidic 70% ethanol. The parasites were dehydrated in a graded ethanol series (70%, 90% and 100%). Parasites were cleared in 50% xylene diluted in ethanol and 100% xylene for 1 min each, and then mounted onto slides with neutral gum, sealed with cover glass, and laid flat to dry. The morphology of the reproductive organs of parasites was observed with a confocal laser scanning microscope (CLSM, Leica TCS SP5, Mannheim, Germany) using an emission wavelength of 488 nm and a scan excitation of 30%. Images were captured and stored at 1024 × 1024 pixels.

### Quantitative analysis of some egg-shell synthesis related genes after piceatannol treatment

Total RNA of 35 mM and 70 mM piceatannol treated *S. japonicum* were extracted as described above. The procedure for qPCR was conducted as described above using primers designed and synthesized by Sangon Biotech Co. Ltd. The PCR primers for *P14* were 5′-GGA CAA CCT CCT CTG GTT CA-3′ and 5′-TTT CTG GAG GTG AAT GAC GA-3′, and for *P48* were 5′-GAG CCC GTT ATA TGC CTC AA-3′ and 5′-CCG GTC GAC ATA CTC CAG AT-3′. The PCR primers for *egg-shell precursor* were 5′-TCA TGG CTT TGC AGA AAC TG-3′ and 5′-GCC AAA TCT CAT CGT GTG AA-3′, and for *FS800* were 5′-TGG AAA CGA AAG TGA TG-3′ and 5′-CTG GAA TTG AAA GGA CC-3′. The PCR primers for *PSMD4* were identical to those described above.

### Statistical analysis

Statistical analysis was performed using GraphPad Prism software (Version 6.0). All data were obtained from three independent experiments, each using triplicate samples and following the same protocol. The statistical significance of the difference between datasets was analyzed using Student’s t-test or one-way analysis of variance (ANOVA) with multiple comparisons of Tukey’s *post-hoc* test. Data are presented as means ± SEM and were considered statistically significant for *P-*values < 0.05.

## Results

### Cloning and sequence characteristics of the *SjTK4* gene

**Fig. 1:**
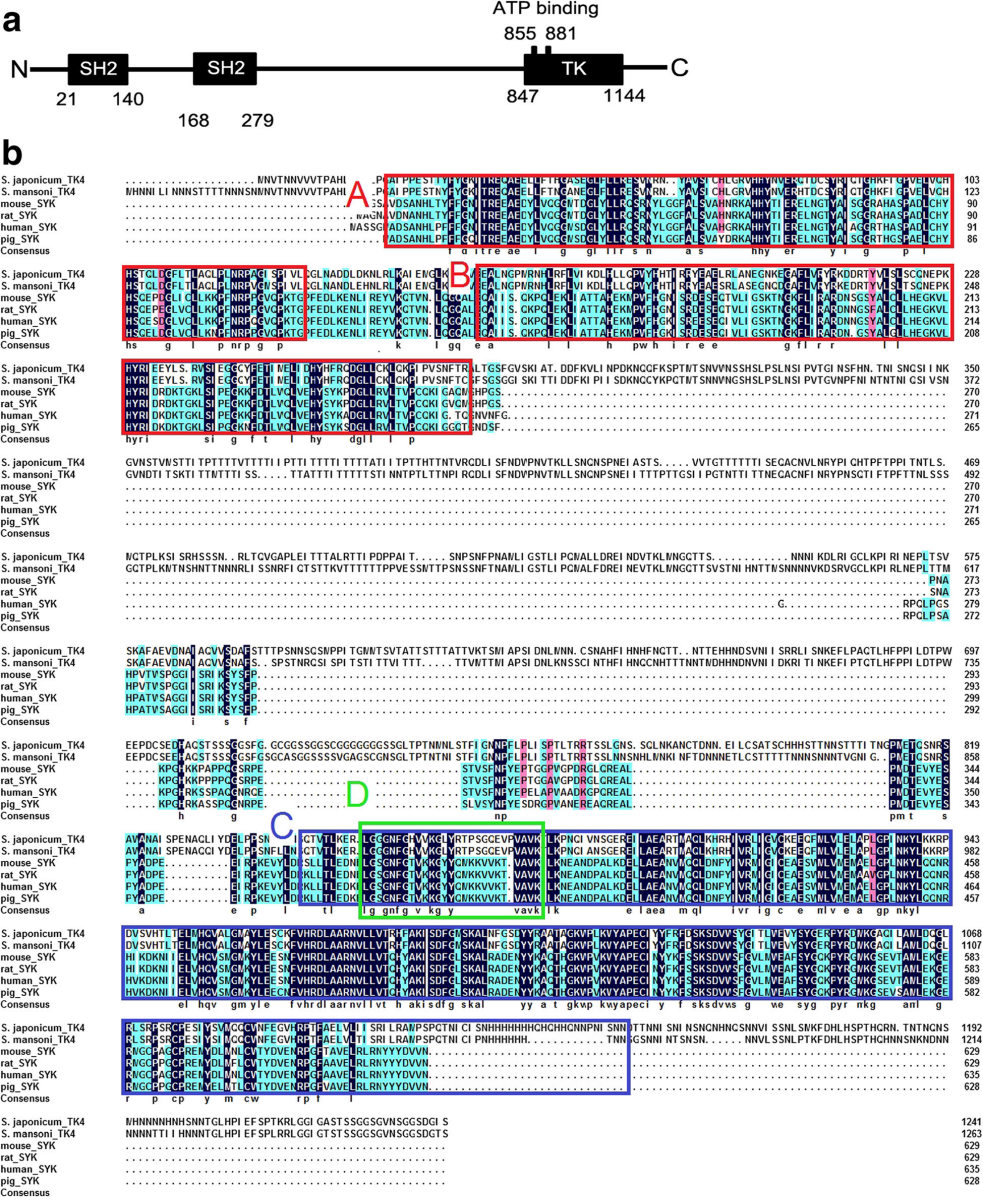
*Schistosoma japonicum* TK4 belongs to the Syk kinase family. **a** Structure of the TK4 domain of *S. japonicum*. The tandem SH2 domain is located at the N-terminus of SjTK4, whereas the catalytic tyrosine kinase domain is located at the C-terminus. They are joined together by a linker region. The numbers in the figure represent the respective sequence coordinates for SjTK4 for each domain. **b** Comparison of amino acid sequences in *S. japonicum* TK4 (SjTK4, APD26308.1) with other organisms: *S. mansoni* (SmTK4, CAD13249.1), *Mus musculus* (mouse Syk, AAA87462.1), *Rattus norvegicus* (rat Syk, NP_036890.1), *Homo sapiens* (human Syk, AAH01645.1), and *Sus scrofa* (pig Syk, NP_001098422.1). The hallmark domains are marked above the alignment, positional information refer to SjTK4. The two red boxes outline the two SH2 domains (5′ SH2 position 21–140, 120aa; 3′ SH2 position, 168–279, 112 aa) and the blue box outlines the TK domain (position 847–1144, 298 aa). The green box delineates a conserved the protein kinase ATP binding domain (position 855–881, 27 aa)

**Fig. 2:**
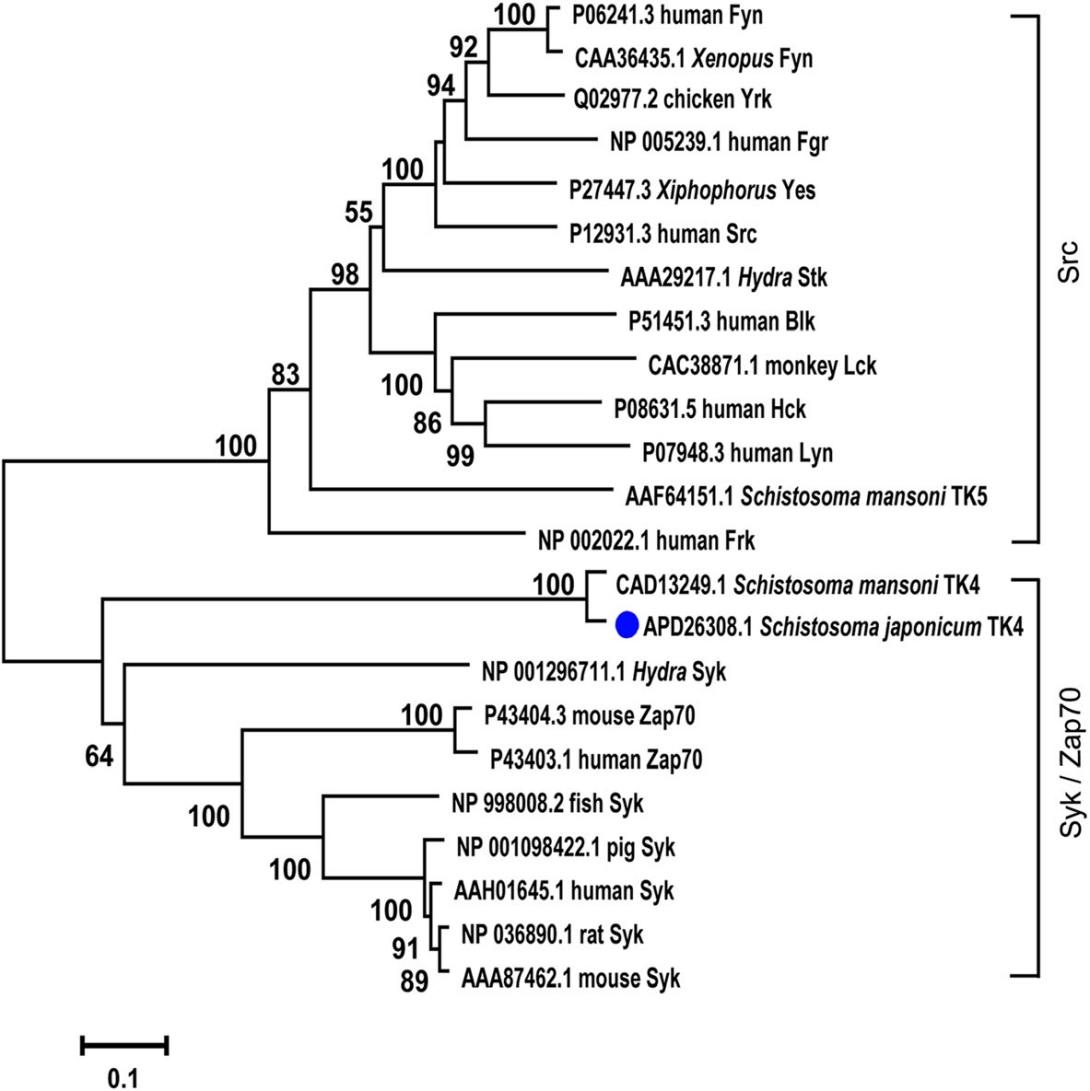
Phylogenetic tree analysis of the TK4 protein in *Schistosoma japonicum*. All of the following sequences were obtained from the National Center for Biotechnology Information (www.ncbi.nlm.nih.gov): human Frk (NP_002022.1), *Hydra* Stk (AAA29217.1), human Fgr (NP_005239.1), human Src (P12931.3), *Xiphophorus* Yes (P27447.3), chicken Yrk (Q02977.2), human Fyn (P06241.3), *Xenopus* Fyn (CAA36435.1), human Blk (P51451.3), human Lyn (P07948.3), human Hck (P08631.5), monkey Lck (*Saimiri sciureus*; CAC38871.1), fish Syk (*Danio rerio*; NP_998008.2), pig Syk (NP_001098422.1), rat Syk (NP_036890.1), mouse Syk (AAA87462.1), human Syk (AAH01645.1), human Zap70 (P43403.1), mouse Zap70 (P43404.3), *Schistosoma mansoni* TK4 (CAD13249.1), *Schistosoma mansoni* TK5 (AAF64151.1), *Hydra* Syk (NP_001296711.1), *Schistosoma japonicum* TK4 (APD26308.1; indicated by the blue dot). The tree is drawn to scale, with branch lengths in the same units as those of the evolutionary distances used to infer the phylogenetic tree. The numbers above the branches indicate the bootstrap values in percentages (of 1000 replicates)

An open reading frame of 3729 bp was amplified, encoding a predicted protein of 1242 amino acids with a predicted molecular weight of 137 kDa (pI 8.37). Information about this protein has been uploaded to the NCBI database (GenBank: APD26308.1). A comparison of the corresponding sequences in the GenBank database from other species indicated that the TK4 amino acid sequence of *S. japonicum* has 76.9% identity and 82.7% similarity to that of *S. mansoni* (CAD13249.1). Using multiple sequence alignment of the amino acid sequences, we found that SjTK4 contains two SH2 domains and one TK domain. Specific conserved motifs within the TK domain were determined to be an ATP-binding region (Fig. 1a, b). Syk-TKs represent a distinct group of cellular TKs in addition to belonging to the large group of Src-like TKs, with the main difference being that Syk-TKs possess two SH2 domains, whereas Src-family members have one SH2 and one SH3 domain [12]. Within the Syk clade of sequences, the SjTK4 cluster was most closely related to the gene products encoded by other species, especially SmTK4 (Fig. 2).

### *SjTK4* gene expression in different developmental stages and tissues of *S. japonicum*

**Fig. 3:**
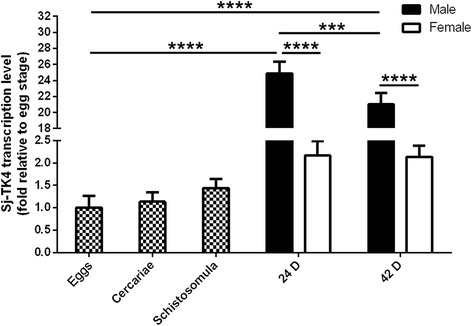
Quantitative analysis of *SjTK4* mRNA expression at various developmental stages and in both sexes. Data represent the mean ± SEM of three independent experiments. Asterisks show statistical differences (***P < 0.001, ****P < 0.0001) tested by one-way ANOVA with multiple comparisons (Tukey’s *post-hoc* test)

**Fig. 4:**
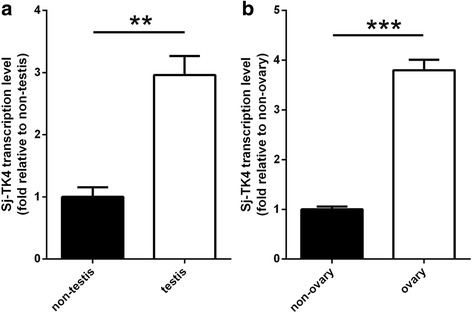
Relative *SjTK4* mRNA expression in the reproductive organs of *Schistosoma japonicum*. Quantitative analysis of ovary and non-ovary tissue in females (**a**) as well as testis and non-testis tissue in males (**b**). Data represent the mean ± SEM of three independent experiments. Asterisks show statistical differences (**P < 0.01, ***P < 0.001) tested by Student’s t-test

Using qPCR, we measured *SjTK4* mRNA levels in eggs, cercariae and schistosomula and in the testis, ovary, non-testis and non-ovary tissues of adult male and female parasites on days 24 and 42. Levels of *SjTK4* were normalized to those for the housekeeping gene *PSMD*. We found that the levels of the *SjTK4* gene were differentially expressed in the various life-cycle stages and between the sexes of *S. japonicum*. The *SjTK4* mRNA expression levels in adult parasites were markedly higher than those in immature parasites, with levels in adult males significantly higher than those in adult females (*F*_(6,14)_ = 538.1, *P* < 0.0001) (Fig. 3). In both male and female parasites, higher amounts of the *SjTK4* gene were expressed in the testis (*t*_(4)_ = 5.703, *P* = 0.0047) and ovary (*t*_(4)_ = 12.96, *P* = 0.0002) relative to non-testis and non-ovary tissues, respectively. (Fig. 4a, b).

### Treatment with piceatannol in vitro affect the reproduction of *S. japonicum*

**Fig. 5:**
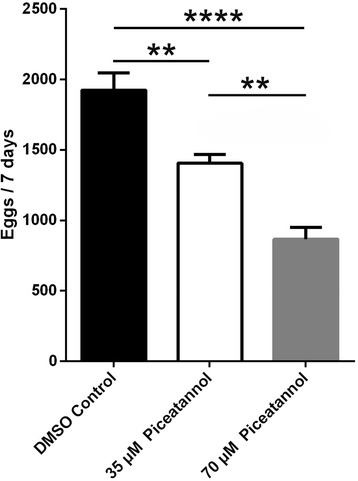
Effects of piceatannol application on spawning in *Schistosoma japonicum*. Worm couples (*n* = 15 couples) were cultured in the absence (DMSO, control) or presence of 35 μM or 70 μM piceatannol. After being cultured in vitro for 7 days, the number of eggs per group was counted. Data represent the mean ± SEM of three independent experiments. Asterisks show statistical differences (**P < 0.01, ****P < 0.0001) tested by one-way ANOVA with multiple comparisons (Tukey’s *post-hoc* test)

We found that parasite viability decreased with increasing inhibitor concentration (data not shown). To analyze the effects of piceatannol on egg production in the paired females, we counted egg numbers and found that the number of eggs (*F*_(2,9)_ = 32.59, *P* < 0.0001) also decreased in a concentration-dependent manner compared to the DMSO-treated group (Fig. 5).

**Fig. 6:**
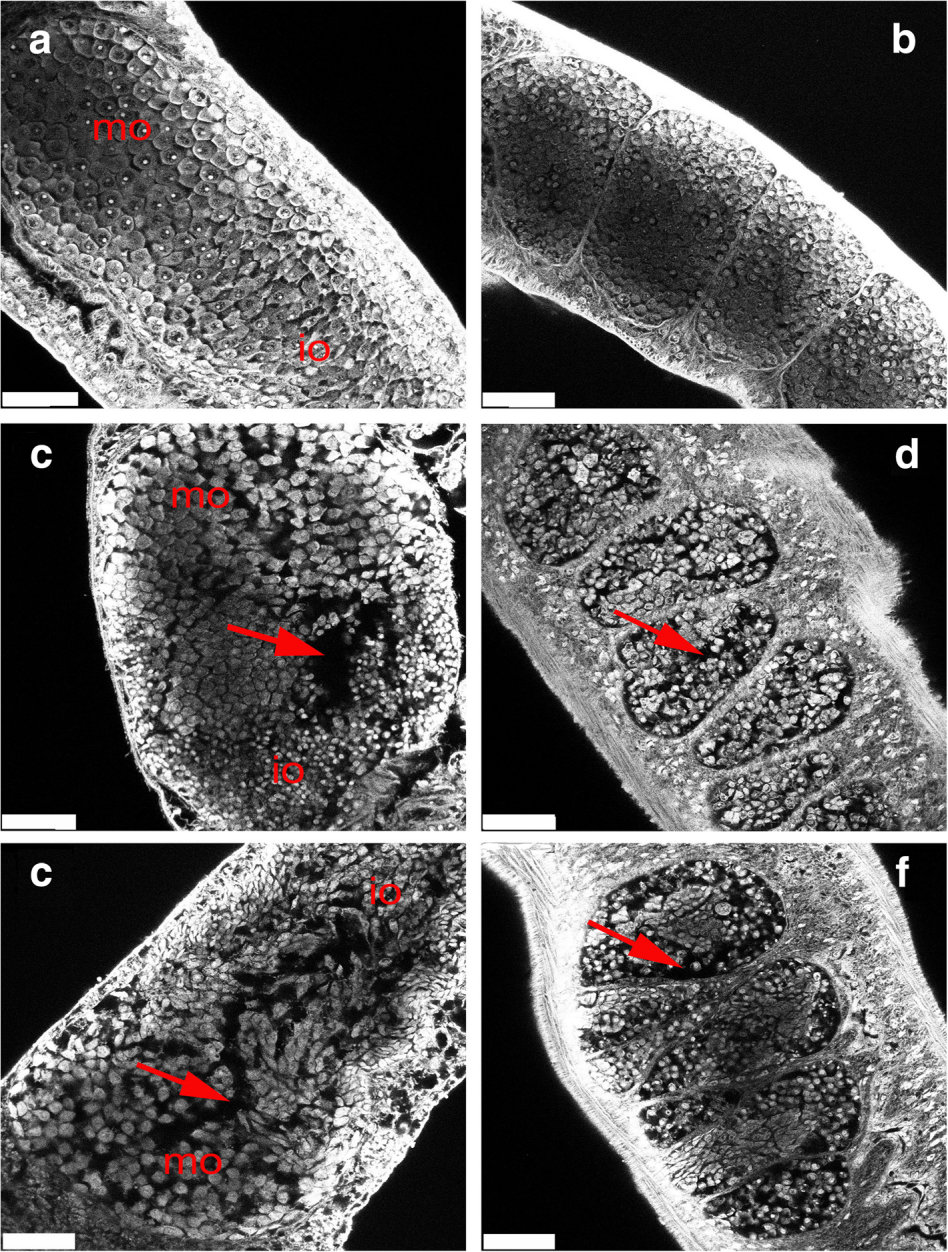
Morphological changes in the testis and ovary of *Schistosoma japonicum* treated with piceatannol. Confocal scanning laser microscope images of carmine-stained whole-mount preparations of *S. japonicum* couples (**a**, **c**, **e**: females; **b**, **d**, **f**: males). **a**, **b** Control parasites treated with DMSO only. **c**, **d** Parasites treated with 35 μM piceatannol for 7 days. **e**, **f** Parasites treated with 70 μM piceatannol for 7 days. *Abbreviations*: io, immature oocytes; mo, mature oocytes; arrows, pore-like structures. *Scale-bars*: 25 μm

After the treatment, parasites were fixed in AFA, stained with carmine, and analyzed using CLSM. In the DMSO-treated group, no morphological anomalies were observed in the testes of the males or the ovaries of the females. The ovaries of DMSO-treated female schistosomes were composed of small immature oocytes in the anterior part and larger primary oocytes in the posterior part (Fig. 6a). The testes of DMSO-treated male schistosomes were composed of several testicular lobes arranged bead-like, and each testicular lobe contained a large number of spermatogonia at different stages and spermatocytes (Fig. 6b). In the group treated with 35 μM piceatannol, the morphology of the whole germ cells in both the testis and ovary were markedly changed. In the ovaries, the sizes of the primary oocytes and immature oocytes were reduced, and the cells of the piceatannol treated groups were not as full as the cells of DMSO group (Fig. 6c). The size of the testicular lobes in the group treated with 35 μM piceatannol was much smaller than that in the DMSO-treated group, and the number of spermatogonia and spermatocytes in the male testis was significantly reduced and more loosely arranged (Fig. 6d). Large pore-like structures could be found in the testis and ovaries of males and females (Fig. 6c-f, arrows). These morphological changes in both females and males were exacerbated after treatment with 70 μM (Fig. 6e-f) and 100 μM piceatannol (data not shown).

### Expression of egg-shell synthesis-related genes

**Fig. 7:**
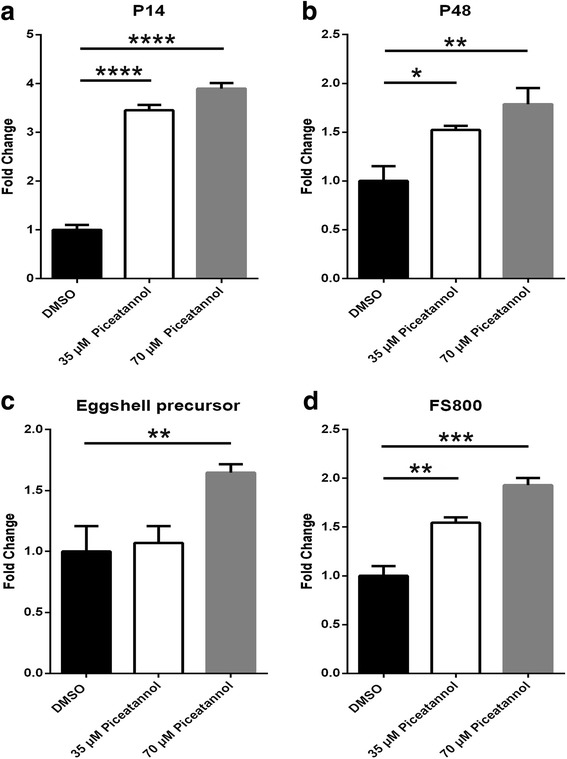
Results of qPCR analyses in *Schistosoma japonicum* treated for 7 days with DMSO or piceatannol. Genes associated with egg-shell synthesis were investigated, including *P14* (**a**), *P48* (**b**), *egg-shell precursor* (**c**), and *FS800* (**d**). Data represent the mean ± SEM of three independent experiments. Asterisks show statistical differences (*P < 0.05, **P < 0.01, ***P < 0.001, ****P < 0.0001) tested by one-way ANOVA with multiple comparisons (Tukey’s *post-hoc* test)

After treating *S. japonicum* with piceatannol in vitro, we performed qPCR analysis of some important egg-shell synthesis-related genes. We found that the expression levels of all these genes, including *P14* (*F*_(2,6)_ = 207.9, *P* < 0.0001), *P48* (*F*_(2,6)_ = 9.309, *P* = 0.0145), eggshell precursor (*F*_(2,6)_ = 5.629, *P* = 0.042)*,* and *FS800* (*F*_(2,6)_ = 34.92, *P* = 0.005), were higher in the piceatannol-treated groups than those in the DMSO-treated group (Fig. 7a-d).

## Discussion

There are several kinds of schistosomes that can infect humans. Although they have a similar life-cycle in their use of mammalian final hosts, these species differ from each other in several respects, such as time of infection and oviposition, the shape and size of eggs, and location of intravascular habitat in mammalian final hosts. These differences between species are important determinants of clinical presentation [31]. It has been confirmed that SmTK4 plays a pivotal role in gametogenesis and affects the development of the testes and ovaries of *S. mansoni* [22]. It is necessary for us to confirm the findings from *S. mansoni* in *S. japonicum*. In the present study, we cloned and sequenced *SjTK4*. Our comparison of the amino acid sequences showed that SjTK4 shares 76.9% identity and 82.7% similarity with SmTK4. Therefore, it is possible that the function of SjTK4 is similar to that of SmTK4. Although significant progress has been made in the study of SmTK4 [22], further exploration of SjTK4 will help us to discover the commonalities between schistosome species. In general, small molecule kinase inhibitors that bind to kinases are broadly categorized into four major classes based on their binding mode, type I, type II, type III and type IV inhibitors [32–34]. We found that human Syk (accession number AAH01645.1) shares 21.3% amino acid identity with SjTK4. The 78.7% amino acid differences between human Syk and SjTK4 may provide a target for selective inhibitors design. However, the sequence similarity within the catalytic domain between SjTK4 and human Syk is 72.9% and the similarity of these two sequences outside the catalytic domain is only 17.2%. This may be difficult for finding novel type I and type II inhibitors, but there is still hope for finding inhibitors of type III and type IV.

The *SjTK4* gene was differentially expressed in the life-cycle stages and sexes of *S. japonicum*. The transcription levels of the *SjTK4* gene in 24 day old male parasites were higher than those at any other stage; this coincides with the time at which paired female schistosomes start laying eggs [35]. The transcription levels of the *TK4* gene in the testis and ovary were higher than those in any other tissue examined; the localization of expression of SjTK4 is consistent with that found in *S. mansoni* [22]. Therefore, we concluded that the SjTK4 signaling pathway may be important for reproductive system maturation and female oviposition after pairing with the male. The higher level of *SjTK4* gene expression that we found in males, compared to females, may be because male parasites possess a greater amount of parenchymatous tissue [12].

In vertebrates and invertebrates, the differentiation of germ cells depends on the reorganization of actin and tubulin cytoskeletons [36–38]. Syk kinases are known to phosphorylate substrates involved in cytoskeleton organization or the components of the cytoskeleton, such as microtubules, directly [19, 39, 40]. In oocytes of marine nemertean worms (*Cerebratulus* spp.), inhibitor studies also showed that Syk kinases play a significant role during oocyte maturation. Using the Syk-kinase specific inhibitor Piceatannol (50–100 mM), a decrease in the maturation of oocytes was observed, indicated by reduced GVBD occurring in early meiosis, and also by reduced MAPK activity [41]. In our present study, after treating *S. japonicum* with piceatannol in vitro, the number of spermatogonia and spermatocytes in the male testis were significantly reduced, and the testicular lobes were significantly smaller than those in the DMSO-treated group. We found large pore-like structures in most of the testis and ovaries of the piceatannol-treated parasites. These results indicate that piceatannol specifically inhibits the proliferation of spermatogonia in males and of primary spermatocytes in females. The proliferation of these cells is essential for the initiation and continuous production of mature germ cells [42]. A reduction in mature germ cells will inevitably reduce the number of eggs. Consequently, the differentiation of the testis and ovaries can have a significant impact on the production and development of eggs. In order to analyze the influence, the number of eggs of paired females was determined after treatment by piceatannol. We found the number of eggs also decreased in a concentration-dependent manner compared to the DMSO-treated group. This suggests that inhibition of SjTK4 signaling pathway may be the alternative for blocking transmission and progression of schistosomiasis.

Many cytoplasmic tyrosine kinases have been identified in *S. mansoni*, such as SmTK3 (Src-like), SmTK4 (Syk-like), SmTK5, SmAbl1/2 and SmTK6 (Src/Abl-like). SmTK3, SmTK4 and SmTK6 form a trimeric kinase complex, which interacts with the receptor tyrosine kinase SmVKR1 to regulate downstream targets [43]. The Src-specific inhibitor Herb A and a TGFβ receptor inhibitor (TRIKI) can block mitosis and egg production in *S. mansoni* and downregulate egg-shell synthesis genes, including *P14*, *FS800*-like, a predicted *egg-shell precursor* protein gene, and tyrosinase 1 [44]. The activity of SmAbl1 is blocked by imatinib (a known Abl-TK inhibitor), which results in reduced egg production, although, unexpectedly, some genes involved in egg-shell synthesis are upregulated [45]. After treating *S. japonicum* with piceatannol in vitro, we found that the levels of genes involved in egg-shell production, including *P14*, *P48*, *egg-shell precursor* and *FS800*, were increased, which was consistent with the results in imatinib-treated *S. mansoni* [45]. Many genes are involved in the complex process of egg-shell formation. The levels of unknown genes that play a critical role in this process may be decreased by piceatannol, whereas the levels of some known genes may be increased to compensate for overall reduced egg formation in piceatannol-treated schistosomes. Schistosome egg production is a complex process that involves not only the participation of different cell types, but also comprises the participation of different organs [35, 46]. Therefore, further study is warranted to determine the molecular mechanisms whereby piceatannol affects egg formation.

## Conclusions

In summary, we investigated the expression profile and provided a preliminary characterization of the *TK4* gene in *S. japonicum*. The results of piceatannol treatment in *S. japonicum* suggest that SjTK4 may exert a regulatory function on the development of the reproductive system, supporting the hypothesis that TK4 inhibitors are potential chemotherapeutics against schistosomiasis. This study provides a good foundation for the design of more specific anti-SjTK4 drugs applicable to anti-schistosome chemotherapy.

## Abbreviations

ANOVA: one-way analysis of variance
CLSM: confocal laser scanning microscopy
Ct: threshold cycle
DMSO: dimethyl sulfoxide
NCBI: National Center for Biotechnology Information
PSMD4: 26S proteasome non-ATPase regulatory subunit 4
PZQ: praziquantel
qPCR: real-time quantitative polymerase chain reaction
SH2: Src homology 2
Syk: spleen tyrosine kinase
TK4: tyrosine kinase 4

## References

[1] Colley DG, Bustinduy AL, Secor WE, King CH (2014). Human schistosomiasis. Lancet.

[2] Zhou XN, Wang LY, Chen MG, Wu XH, Jiang QW, Chen XY, et al. The public health significance and control of schistosomiasis in China - then and now. Acta Trop 2005;96(2-3):97–105. 10.1016/j.actatropica.2005.07.005 16125655

[3] Zhou YB, Liang S, Jiang QW (2012). Factors impacting on progress towards elimination of transmission of schistosomiasis japonica in China. Parasit Vectors.

[4] Pinto-Almeida A, Mendes T, de Oliveira RN, Correa Sde A, Allegretti SM, Belo S (2016). Morphological characteristics of *Schistosoma mansoni* PZQ-resistant and -susceptible strains are different in presence of praziquantel. Front Microbiol.

[5] Doenhoff MJ, Cioli D, Utzinger J (2008). Praziquantel: mechanisms of action, resistance and new derivatives for schistosomiasis. Curr Opin Infect Dis.

[6] Cogswell AA, Kommer VP, Williams DL (2012). Transcriptional analysis of a unique set of genes involved in *Schistosoma mansoni* female reproductive biology. PLoS Negl Trop Dis.

[7] Wilson MS, Mentink-Kane MM, Pesce JT, Ramalingam TR, Thompson R, Wynn TA (2007). Immunopathology of schistosomiasis. Immunol Cell Biol.

[8] Nady S, Shata MT, Mohey MA, El-Shorbagy A. Protective role of IL-22 against *Schistosoma mansoni* soluble egg antigen-induced granuloma in vitro. Parasite Immunol. 2017;39(1):pim.12392. 10.1111/pim.12392 27741351

[9] Diao Y, Hua M, Shao Y, Huang W, Liu M, Ren C (2015). Preliminary characterization and expression of vasa-like gene in *Schistosoma japonicum*. Parasitol Res.

[10] Jiang YY, Yuan ZY, Xu YX, Zang W, Cao JP, Wang Y, et al. [Molecular characteristics and RNA interference efficiency of *Schistosoma japonicum* Sj79 gene.] Zhongguo xue xi chong bing fang zhi za zhi. 2015;27(3):273–6 (In Chinese). 26510359

[11] Ta N, Feng XG, Deng LL, ZQ F, Hong Y, Liu JM (2015). Characterization and expression analysis of Wnt5 in *Schistosoma japonicum* at different developmental stages. Parasitol Res.

[12] Knobloch J, Winnen R (2002). Quack M, Kunz W, Grevelding CG. A novel Syk-family tyrosine kinase from *Schistosoma mansoni* which is preferentially transcribed in reproductive organs. Gene.

[13] Mocsai A, Ruland J, Tybulewicz VL (2010). The SYK tyrosine kinase: a crucial player in diverse biological functions. Nat Rev Immunol.

[14] Deng GM, Kyttaris VC, Tsokos GC (2016). Targeting Syk in autoimmune rheumatic diseases. Front Immunol.

[15] Page TH, Smolinska M, Gillespie J, Urbaniak AM, Foxwell BMJ (2009). Tyrosine kinases and inflammatory signalling. Curr Mol Med.

[16] Inatome R, Yanagi S, Takano T, Yamamura HA (2001). Critical role for Syk in endothelial cell proliferation and migration. Biochem Biophys Res Commun.

[17] Fluck M, Zurcher G, Andres AC, Ziemiecki A (1995). Molecular characterization of the murine Syk protein tyrosine kinase cDNA, transcripts and protein. Biochem Biophys Res Commun.

[18] Tsujimura T, Yanagi S, Inatome R, Takano T, Ishihara I, Mitsui N (2001). Syk protein-tyrosine kinase is involved in neuron-like differentiation of embryonal carcinoma P19 cells. FEBS Lett.

[19] Coopman PJ, Mueller SC (2006). The Syk tyrosine kinase: a new negative regulator in tumor growth and progression. Cancer Lett.

[20] Ferrante AW Jr, Reinke R, Stanley ER. Shark, a Src homology 2, ankyrin repeat, tyrosine kinase, is expressed on the apical surfaces of ectodermal epithelia. Proc Natl Acad Sci USA. 1995;92(6):1911–5. 10.1073/pnas.92.6.1911 PMC42392 7892198

[21] Steele RE, Stover NA, Sakaguchi M (1999). Appearance and disappearance of Syk family protein-tyrosine kinase genes during metazoan evolution. Gene.

[22] Beckmann S, Buro C, Dissous C, Hirzmann J, Grevelding CG (2010). The Syk kinase SmTK4 of *Schistosoma mansoni* is involved in the regulation of spermatogenesis and oogenesis. PLoS Pathog.

[23] Elisabeth G, Alexandre G, Christine H, Ivan I, Ron DA, Amos B (2003). ExPASy: the proteomics server for in-depth protein knowledge and analysis. Nucleic Acids Res.

[24] Rice P1, Longden I, Bleasby A (2000). EMBOSS: the European molecular biology open software suite. Trends Genet.

[25] Quevillon E, Silventoinen V, Pillai S, Harte N, Mulder N, Apweiler R, Lopez R (2005). InterProScan: protein domains identifier. Nucleic Acids Res.

[26] Tamura K, Peterson D, Peterson N, Stecher G, Nei M, Kumar S. MEGA: Molecular Evolutionary Genetics Analysis using maximum likelihood, evolutionary distance, and maximum parsimony methods. Mol Biol Evol. 2011;28(10):2731–9. 10.1093/molbev/msr121 PMC3203626 21546353

[27] Wang J, Yu Y, Shen H, Qing T, Zheng Y, Li Q (2017). Dynamic transcriptomes identify biogenic amines and insect-like hormonal regulation for mediating reproduction in *Schistosoma japonicum*. Nat Commun.

[28] Zhu L, Zhao J, Wang J, Hu C, Peng J, Luo R (2016). MicroRNAs are involved in the regulation of ovary development in the pathogenic blood fluke *Schistosoma japonicum*. PLoS Pathog.

[29] Milligan JN, Jolly ER (2012). Identification and characterization of a Mef2 transcriptional activator in schistosome parasites. PLoS Negl Trop Dis.

[30] Liu S, Cai P, Hou N, Piao X, Wang H, Hung T (2012). Genome-wide identification and characterization of a panel of house-keeping genes in *Schistosoma japonicum*. Mol Biochem Parasitol.

[31] Ferrari TC, Moreira PR (2011). Neuroschistosomiasis: clinical symptoms and pathogenesis. Lancet Neurol.

[32] Gavrin LK, Saiah E (2013). Approaches to discover non-ATP site kinase inhibitors. Medchemcomm.

[33] Simard JR, Kluter S, Grutter C, Getlik M, Rabiller M, Rode HB (2009). A new screening assay for allosteric inhibitors of cSrc. Nat Chem Biol.

[34] Ohren JF, Chen H, Pavlovsky A, Whitehead C, Zhang E, Kuffa P (2004). Structures of human MAP kinase kinase 1 (MEK1) and MEK2 describe novel noncompetitive kinase inhibition. Nat Struct Mol Biol.

[35] Kunz W (2001). Schistosome male-female interaction: induction of germ-cell differentiation. Trends Parasitol.

[36] Xiao X, Yang WX (2007). Actin-based dynamics during spermatogenesis and its significance. J Zhejiang Univ Sci B.

[37] Mansir A, Justine JL (1998). The microtubular system and posttranslationally modified tubulin during spermatogenesis in a parasitic nematode with amoeboid and aflagellate spermatozoa. Mol Reprod Dev.

[38] Sun QY, Schatten H (2006). Regulation of dynamic events by microfilaments during oocyte maturation and fertilization. Reproduction.

[39] Peters JD, Furlong MT, Asai DJ, Harrison ML, Geahlen RL (1996). Syk, activated by cross-linking the B-cell antigen receptor, localizes to the cytosol where it interacts with and phosphorylates alpha-tubulin on tyrosine. J Biol Chem.

[40] Faruki S, Geahlen RL, Asai DJ (2000). Syk-dependent phosphorylation of microtubules in activated B-lymphocytes. J Cell Sci.

[41] Stricker SA, Smythe TL (2006). Differing mechanisms of cAMP- *versus* seawater-induced oocyte maturation in marine nemertean worms II. The roles of tyrosine kinases and phosphatases. Mol Reprod Dev.

[42] Braydich-Stolle L, Kostereva N, Dym M, Hofmann MC (2007). Role of Src family kinases and N-Myc in spermatogonial stem cell proliferation. Dev Biol.

[43] Beckmann S, Hahnel S, Cailliau K, Vanderstraete M, Browaeys E, Dissous C (2011). Characterization of the Src/Abl hybrid kinase SmTK6 of *Schistosoma mansoni*. J Biol Chem.

[44] Buro C, Oliveira KC, Lu Z, Leutner S, Beckmann S, Dissous C, et al. Transcriptome analyses of inhibitor-treated schistosome females provide evidence for cooperating Src-kinase and TGFbeta receptor pathways controlling mitosis and egg-shell formation. PLoS Pathog. 2013;9(6):e1003448. 10.1371/journal.ppat.1003448 PMC3681755 23785292

[45] Buro C, Beckmann S, Oliveira KC, Dissous C, Cailliau K, Marhofer RJ (2014). Imatinib treatment causes substantial transcriptional changes in adult *Schistosoma mansoni in vitro* exhibiting pleiotropic effects. PLoS Negl Trop Dis.

[46] Popiel I, Basch PF (1984). Reproductive development of female *Schistosoma mansoni* (Digenea: Schistosomatidae) following bisexual pairing of worms and worm segments. J Exp Zool.

